# Proceedings of the 3rd PSMR Conference on PET/MR and SPECT/MR

**DOI:** 10.1186/2197-7364-1-S1-A1

**Published:** 2014-07-29

**Authors:** George Loudos, Alberto del Guerra, N Jon Shah

**Affiliations:** Technological Educational Institute of Athens, Kragujevac, Greece; University of Pisa, Kragujevac, Italy; Forschungszentrum Juelich GmbH, Kragujevac, Germany

The contributions of the present European Journal of Nuclear Medicine and Molecular Imaging Physics Proceedings issue “PSMR2014 – 3^rd^ International Conference on PET-MR and SPECT-MR” give a representative overview of oral and poster presentations at the third Conference on PET-MR and SPECT-MR. PSMR2014 took place in Kos Island (Greece) in May 19–21 and was organized in cooperation of the Technological Educational Institute of Athens, the Pisa University-INFN, the Forschungszentrum Juelich GmbH and the COST Action PET-MRI TD-1007.

COST is an intergovernmental framework for European Cooperation in Science and Technology, allowing the coordination of nationally-funded research on a European level. The COST Action TD1007: “Bimodal PET-MRI molecular imaging technologies and applications for in vivo monitoring of disease and biological processes”, was founded by COST Office in 2011 and aims to provide a framework for collaboration between European institutions that have significant experience and research activity in scientific fields that are complementary for PET/MRI research.

Like the PSMR2012 and PSMR2013, PSMR2014 covered all technological aspects and applications of PET/MR and SPECT/MR systems. The sessions entitled Advances in MR-PET instrumentation; Advances in MR-PET software and quantification; Whole-body MR-PET; Dedicate organ MR-PET; Advances in MR-SPET; Bimodal tracers and Preclinical MR-PET applications. In addition, latest industrial developments were reported in a dedicated industrial session. The keynote lecture was given by Prof. Andrew Maudsley (USA) on “MR Spectroscopy”. There were 55 oral and 55 poster contributions. More than 180 registered participants – physicists, engineers, radiochemists, technologists and physicians – enjoyed the program comprising all the different aspects of the new bimodal imaging modalities and the lively discussions following the presentations and during the breaks.

Please find more details on PSMR2014 with links to the previous workshops at http://www.mr-pet.eu/psmr14.html.

